# Effectiveness of the Pasos Adelante Chronic Disease Prevention and Control Program in a US-Mexico Border Community, 2005-2008

**Published:** 2011-12-15

**Authors:** Lisa K. Staten, Christina Cutshaw, Kerstin Reinschmidt, Rosie Stewart, Denise J. Roe, Christopher Davidson

**Affiliations:** Department of Public Health, School of Medicine, Indiana University; Mel and Enid Zuckerman College of Public Health, University of Arizona, Tucson, Arizona; Mel and Enid Zuckerman College of Public Health, University of Arizona, Tucson, Arizona; Mel and Enid Zuckerman College of Public Health, University of Arizona, Tucson, Arizona; Mel and Enid Zuckerman College of Public Health, University of Arizona, Tucson, Arizona; Mel and Enid Zuckerman College of Public Health, University of Arizona, Tucson, Arizona

## Abstract

**Introduction:**

Pasos Adelante is a lifestyle intervention program facilitated by community health workers (promotores) targeting chronic disease prevention and control in Mexican Americans. Initial studies of Pasos Adelante indicated significant improvements in self-reported nutrition and physical activity. This study examined whether Pasos Adelante participants living in a US border community showed improvements in selected physiological measures after participating in the program and whether changes were maintained at 3-month follow-up.

**Methods:**

The program took place in 12-week sessions from January 2005 to May 2008 and included walking groups and education targeting nutrition and physical activity. Questionnaires, anthropometric measures, and laboratory tests were conducted at baseline (n = 305), conclusion of program (n = 254), and 3-month follow-up (n = 221).

**Results:**

Participants demonstrated decreases in body mass index (*P* = .04), waist and hip circumference (*P* < .001), diastolic and systolic blood pressure (*P* < .001), and total cholesterol (*P* = .008) from baseline to program conclusion. No values worsened significantly between program conclusion and follow-up, except systolic blood pressure. Glucose levels improved between conclusion and follow-up (*P* = .01).

**Conclusion:**

These results support the initial findings of improvements in participants' self-reported physical activity and nutrition patterns through changes in objective measures. This evidence-based program demonstrates the potential for a promotores-facilitated chronic disease prevention and control program to improve physical health and targets both primary and secondary prevention in Hispanic communities and organizations.

## Introduction

Mexican American weight patterns were first documented in 1982 to 1984, when an estimated 60% of adults were overweight or obese compared to 44% for non-Hispanic whites (1). By 2007 to 2008, the proportion increased to 79% of Mexican Americans compared to 67% of non-Hispanic whites (2). Overweight and obesity rates put Mexican Americans at increased risk for developing chronic diseases such as cardiovascular disease (CVD) and diabetes, the first and fifth leading causes of death for US Hispanics (3).

The rate of diabetes among Hispanics is more than 50% higher than that of non-Hispanic whites (4). By 2050, diabetes rates among Hispanics are expected to increase by 481% from 2005 levels (5). Mexican American adults living along the US-Mexico border experience double the US rate of diabetes (6,7). Hospital discharge data from US border states found higher diabetes-related discharge rates for Hispanics compared to non-Hispanic whites living in the area and Hispanics from non-border communities (8).

Randomized, controlled trials demonstrate that lifestyle changes can delay and even prevent the onset of type 2 diabetes (9,10) and CVD (11). Many Hispanic women do not recognize CVD as a leading cause of death (12), and many do not believe they can prevent either CVD or diabetes. Although risk factors are consistent for both diabetes and CVD, few programs target both diseases simultaneously. Pasos Adelante is a lifestyle intervention program targeting chronic disease prevention and control among Mexican Americans. Initial studies of Pasos Adelante indicated significant improvements in subjective measures of self-reported nutrition and physical activity (13) but did not include more objective measures. To determine whether the intervention leads to more objective changes, we conducted a follow-up study to examine whether Pasos Adelante participants living in a US border community experienced improvements in select physiological measures after participating in the program and whether changes were maintained 3 months after the program ended.

## Methods

This was a quasi-experimental pre-test, post-test study, with follow-up, to test whether participation in the 12-week Pasos Adelante program would result in significant physiological improvements among residents of 1 US-Mexico border community. Pasos Adelante is a culturally tailored 12-week curriculum developed in collaboration with community and academic partners and based on the National Heart, Lung, and Blood Institute's Su Corazón, Su Vida (1st edition) 9-week curriculum (14). The intervention used multiple levels of the socioecological model (15) by focusing strategies on intrapersonal (social cognitive theory), interpersonal (social support), and broader community (community empowerment) levels. The program included didactic sessions targeting chronic disease prevention and control through a focus on physical activity and nutrition and was facilitated by community health workers, or promotores as they are known in the Hispanic community. In addition, the curriculum incorporated walking groups to target behavioral capability and self-efficacy, which are key constructs of social cognitive theory. Recommended best practices from the *Guide to Community Preventive Services* were incorporated in Pasos Adelante: social support in community settings (recommended for diabetes management, nutrition, and physical activity) and group education (recommended for diabetes management) (16,17). In addition, essential elements of the Diabetes Prevention Program (9) were incorporated.

Details of the development and contents of the curriculum are available (13). The curriculum is available in Spanish and English on the Arizona Prevention Research Center website (http://azprc.arizona.edu).

### Study setting

Participants in the Pasos Adelante program were residents of Douglas, Arizona. Douglas is located in the southeast corner of the state approximately 50 miles west of the New Mexico border and directly on the border between Arizona and Sonora, Mexico. Historically, Douglas was known for its smelter operations that processed copper from nearby mines until the operations shut down in 1987. According to the 2000 US Census, the population is approximately 86% Hispanic, and approximately 9,532 people are aged 18 years or older (18).

The primary employers in Douglas are the federal government (US Border Patrol), state government (prison and local government agencies), and private industry (eg, Safeway, Walmart). Despite the changing economic conditions and national spotlight because of illegal immigration and drug trafficking, the community has worked hard to create a healthy environment for its residents. In 1996, community members became concerned about perceived high rates of diabetes in their community and approached University of Arizona College of Public Health faculty (6,19). Since then, the community has worked in partnership with university personnel to address diabetes prevention and control.

### Recruitment

Three promotoras (female promotores) who had lived in the community 20 to 35 years recruited participants and facilitated the intervention. Two of the 3 promotoras had facilitated the Pasos Adelante program in their community for a 1-year pilot phase. The third promotora shadowed the more experienced promotoras before facilitating sessions. All 3 promotoras attended a 1-week, half-day training for facilitating the Su Corazón, Su Vida curriculum at a national conference. Ongoing trainings included quarterly refresher trainings and local and national conferences.

Using opportunistic methods, promotoras recruited cohorts of approximately 30 participants by attending public events such as health fairs and Rotary club meetings. Over time, waiting lists were established for the program, and most recruitment occurred through referrals from former participants. The only inclusion criteria were that participants be aged 18 or older and consider themselves residents of the Douglas community. Participants for this project were recruited from January 2005 through February 2008. One cohort completed the program before the next cohort was recruited.

### Study design

All study protocols were approved by the University of Arizona institutional review board. Interviewer training consisted of multiple day-long reviews of the questionnaire, including discussions of the intent of each question to ensure that all personnel fully understood the questions. A training manual documenting the intention of each question was developed and provided to all staff conducting interviews. Training emphasized the need to strictly follow questions as worded and appropriate prompts for questions. New staff observed multiple administrations of the interview by an experienced interviewer. They were then observed for their first few interviews to ensure consistency.

Baseline measures were taken between 1 month before participation in the Pasos Adelante program and the second week of class. Participants gave their informed consent before completing a baseline survey, having their anthropometric measures taken, and being referred for a fasting blood draw. Interviews and anthropometric measures were conducted by project personnel at a local church, the site of the Pasos Adelante classes. Anthropometric measures took place in the church kitchen in a semiprivate location. Participants were referred for a fasting blood draw at the local hospital. Measures were repeated within 6 weeks after the conclusion of the program and again at a 3-month follow-up. The promotoras who facilitated Pasos Adelante conducted interviews at baseline did not interview the participants at the conclusion of the program or 3-month follow-up assessments to reduce the potential for bias. These data were collected by other project staff. The promotoras took the anthropometric and physiological measures at each assessment but did not have access to prior results.

### Measures

The project questionnaire consisted of 70 questions that assessed demographic characteristics, physical activity, dietary practices, and medical history, including chronic disease, access to medical care, depression symptoms, and health-related quality of life. Questionnaires were interviewer-administered in the participants' preferred language, either Spanish or English, by bilingual personnel on paper forms. Interview length averaged 45 minutes (range, 20-90 min).

One of the authors (L.S.) trained project staff members to conduct standardized height, weight, waist and hip circumference, blood pressure, and pulse measurements (20). Height was measured to the nearest 0.5 cm using a stadiometer; weight was measured to the nearest 1 kg. Both height and weight were measured using an Eye Level Beam Scale (Cardinal Scale Manufacturing Co, Detecto, Missouri); participants wore light clothing and removed their shoes. Two blood pressure measures were also taken 5 minutes apart using the same arm. Waist and hip circumferences were measured to the nearest 0.1 cm. With the exception of blood pressure, all measures were obtained in triplicate; a fourth measure was taken if the initial measures differed by more than a specified amount (>1.0 cm for height, >0.5 kg for weight, >5 mm Hg for systolic or diastolic blood pressure, >1.5 cm for waist and hip measures) (21). Measures were repeated at baseline, conclusion of the program, and 3-month follow-up. Measurement periods varied slightly because of scheduling around holidays. Inter- and intra-staff reliability in taking measures was assessed annually. Staff members whose measures were outside of acceptable reliability ranges were not allowed to conduct participant measurements until they were able to complete measurements that fell within acceptable ranges.

Fasting blood samples were analyzed for total serum cholesterol, blood glucose, and triglyceride levels at a single certified laboratory. Initial funding was for a baseline and 3-month follow-up but did not allow for blood measures at the conclusion of the program. Funding was obtained in the second year of the study for all 3 measurement periods. Therefore, 102 participants were not eligible for blood draws at all 3 assessment periods.

### Statistical analyses

We analyzed selected demographic variables for all participants with baseline assessments. To assess the extent of study completion bias, baseline demographic variables for study completers were compared with baseline variables for participants who did not complete all 3 assessment periods using χ^2^ or Fisher exact tests. We compared the mean baseline age for completers and noncompleters using the *t* test.

Anthropometric and laboratory measures were summarized using the mean, standard deviation, minimum, and maximum values. The mean of the 3 measures taken at each assessment period for each anthropometric variable was used as the point estimate for each participant. If a fourth measure was collected, the mean of the 3 measures with the smallest standard deviation was used as the point estimate.

A linear mixed-effects model was used to assess the effect of time (independent variable) on each physiological measure. The model included all participants with at least a baseline measure regardless of the number of additional measures. Because the study population was homogeneous for sex, age, ethnicity, and language preference, we did not pursue models to control for these demographic variables.

To assess movement between body mass index (BMI) categories, we collapsed the categories of normal/overweight and obese/extremely obese and assessed the differences in proportions in each category from baseline to conclusion of the program and from conclusion to 3-month follow-up. The McNemar test for paired differences was used to test the null hypothesis that the proportions of patients who switched categories were equal. Significance was set at *P* < .05. All statistical procedures were conducted with SAS version 9.2 (SAS Institute, Inc, Cary, North Carolina).

## Results

Between January 2005 and February 2008, 327 people enrolled in the Pasos Adelante program. Twenty-two (7%) had participated in another intervention targeting diabetes prevention or control before participating in Pasos Adelante and were excluded from statistical analyses presented in this article (Figure). Of the 305 remaining participants, 255 (84%) completed the assessment at the conclusion of program, and 221 (73%) completed the 3-month follow-up assessments (questionnaire and anthropometric measures); 217 (71%) participants completed all 3 assessments. Participants were primarily Hispanic women who were born in Mexico, preferred speaking Spanish, were married, and were not educated beyond high school (Table 1). More than half of participants were covered by health insurance, including Medicare. More than 61% of participants reported a family history of diabetes. Approximately 21% of participants reported being diagnosed with diabetes, and approximately 80% of these had been diagnosed for at least 1 year. Approximately 86% were overweight or obese at baseline. Those who completed all 3 assessments were older than the noncompleters (*P* < .001), and more had health insurance (*P* = .045), but these 2 groups did not differ for any other demographic variables or health indicators (data not shown).

**Figure.**
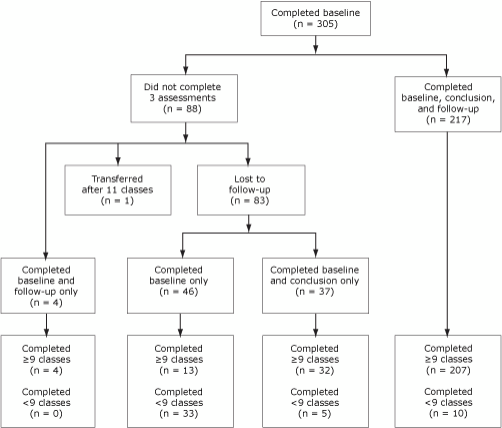
Participant flow, Pasos Adelante program, Douglas, Arizona, 2005-2008.

Mean values for all anthropometric measures, total and HDL cholesterol, and glucose show a downward trend from baseline to program conclusion and additional declines at follow-up in BMI and hip circumference (Table 2). Time of assessment was significantly associated with all anthropometric variables except pulse (Table 3). BMI, waist and hip circumferences, waist-to-hip ratio, and both diastolic and systolic blood pressures were significantly lower at the conclusion of the program compared to baseline. No significant change in anthropometric variables was observed between the conclusion of the program and the 3-month follow-up, with the exception of systolic blood pressure. Time of assessment was also significantly associated with total cholesterol and blood glucose levels. Total cholesterol was significantly lower at the conclusion of the program compared to baseline and was maintained at 3-month follow-up. Glucose levels dropped significantly between the conclusion of the program and 3-month follow-up. Our results suggest that the reductions in anthropometric measures from the conclusion of the program through the 3-month follow-up were maintained.

The percentage of participants who moved from the obese or extremely obese category to the overweight or normal category was not significant at the conclusion of the program but had become significant by follow-up (*P* = .03) (data not shown).

## Discussion

Pasos Adelante participants demonstrated significant improvement in physiological measures linked to diabetes and cardiovascular risk factors after participating in the 12-week promotora-facilitated program that combines interactive educational sessions with walking groups. These changes support earlier findings from participants in 2 other border communities of significant increases in physical activity and consumption of healthier food based on self-report (13).

With the exception of systolic blood pressure, none of the physiological measures rebounded to the participants' baseline values; however, a pattern of slightly increasing values was evident. These results suggest that many of the behaviors learned during the program may be maintained, but that future studies are needed to examine long-term effects. Booster sessions or other ongoing support may be needed to maintain and promote continued improvements of biological risk factors.

The Pasos Adelante program targeted the general adult community to promote primary and secondary prevention and to appeal to the broader community. The broad inclusion criteria (being 18 or older and a resident of Douglas) allowed for participation of friends and family members, all of whom were the focus of the intervention but made assessment challenging. Some of the participants enrolled in the program did not have overt health risks such as a diagnosed chronic disease or symptoms, and entered the program with all normal laboratory values, but 86% of them were overweight or obese at baseline.

Our study has several limitations. Because the study design lacked a randomized control group and called for opportunistic recruitment methods, alternative explanations for change in the outcomes of interest must be considered. First, opportunistic recruitment may have resulted in a selection bias for participants. However, the goal of this intervention was to test the effectiveness of a behavior change intervention in a real-world setting such as might occur if an agency offered the program to the general public. The overrepresentation of older women in health promotion programs is frequently documented. Second, participants may have engaged in other nutrition and physical activity–focused interventions concurrently with Pasos Adelante, but our knowledge about the program offerings in this community suggests that this explanation is unlikely. A secular trend toward decreasing body measures over a 12-week period was theoretically possible, but our findings are in contrast to general secular trends. The US trend for weight has been increasing, not decreasing as seen in our participants. For example, between data collected for 1988 to 1994 and those for 2008 the prevalence of obesity increased among Mexican American men from 24% to 36% and among Mexican American women from 35% to 45% (22). The most optimistic findings demonstrate no change, not a decrease in the physiological measures demonstrated here.

The Pasos Adelante program demonstrated results that are comparable to other programs facilitated by community health workers that targeted high-risk people. For example, Balcazar and colleagues conducted a randomized controlled trial targeting people with at least 1 CVD risk factor using the Su Corazón, Su Vida curriculum (the basis for the Pasos Adelante curriculum) during a 4-month period (23). The participants had similar demographic characteristics to our sample in that they were from a US-Mexico border community, predominantly female with a mean age of 54; 67% had a family history of diabetes, and most were either bilingual or spoke Spanish. In unadjusted results, the experimental group had significant decreases in weight, blood pressure, and cholesterol while the control group also had decreased blood pressure but increased hemoglobin A1c and waist circumference.

In conclusion, participants in the Pasos Adelante program demonstrated significant decreases in key risk factors for CVD and diabetes, many of which were maintained 12 weeks after completion of the program. These results support the initial findings of improvements in self-reported physical activity and nutrition patterns (13). This program demonstrates the potential for promotores-facilitated chronic disease prevention and control programs to affect physical health and provides Hispanic communities and organizations with a choice for an evidence-based program that targets both primary and secondary prevention.

## Figures and Tables

**Table 1:** Demographic and Health Status Characteristics of Participants, Pasos Adelante Program, Douglas, Arizona, 2004-2009

Characteristic	No. of Baseline Participants (%) (n = 305)	No. of Completers (%) (n = 217)	No. of Noncompleters (%) (n = 88)	*P* Value^a^
**Demographic**				
**Age, mean (SD), y**	52.2 (13.9)	54.4 (12.5)	46.7 (15.6)	<.001
**Hispanic ethnicity**	305 (100)	217 (100)	88 (100)	NA
**Female sex**	279 (91.5)	200 (92.2)	79 (89.7)	.50
**Marital status**				
Married	201 (65.9)	144 (66.4)	57 (64.8)	.16
Single/divorced/separated	74 (24.3)	48 (22.1)	26 (29.5)	
Widowed	30 (9.8)	25 (11.5)	5 (5.7)	
**Education**				
Some elementary	29 (9.5)	20 (9.2)	9 (10.2)	.37
Completed elementary	47 (15.4)	39 (18.0)	8 (9.1)	
Some high school	103 (33.8)	69 (31.8)	34 (38.6)	
Completed high school	71 (23.3)	51 (23.5)	20 (22.7)	
Post high school	55 (18.0)	38 (17.5)	17 (19.3)	
**Currently employed**	103 (33.8)	68 (31.3)	35 (39.8)	.16
**Health insurance coverage**	166 (54.4)	126 (58.1)	40 (45.5)	.045
**Preferred speaking language**				
English	9 (3.0)	5 (2.3)	4 (4.5)	.28^b^
Spanish	274 (89.8)	193 (88.9)	81 (92.0)	
No preference	22 (7.2)	19 (8.8)	3 (3.4)	
**Preferred reading language**				
English	16 (5.2)	12 (5.5)	4 (4.6)	>.99^b^
Spanish	274 (89.8)	191 (88.0)	83 (94.3)	
No preference	15 (4.9)	14 (6.5)	1 (1.1)	
**Length of residence in community, y**				
≤5	77 (25.2)	48 (22.1)	29 (33.0)	.06
6-10	41 (13.4)	27 (12.4)	14 (15.9)	
>10	187 (61.3)	142 (65.4)	45 (51.1)	
**Born in Mexico**	271 (88.9)	191 (88.0)	80 (90.9)	.47
**Health status**				
**Family member diagnosed with diabetes**	187 (61.3)	131 (60.3)	56 (64.4)	.55
**Diagnosed with diabetes**	66 (21.6)	46 (21.2)	20 (22.7)	.77
**Length of time with diabetes, y^c^**				
<1	11 (16.7)	9 (19.6)	2 (10.0)	.35
1-5	31 (47.0)	23 (50.0)	8 (40.0)	
6-10	12 (18.2)	6 (13.0)	6 (30.0)	
>10	12 (18.2)	8 (17.4)	4 (20.0)	
**Body mass index, kg/m^2^**				
<25.0 (normal)	42 (13.8)	27 (12.4)	15 (17.1)	.12
25.0-29.9 (overweight)	98 (32.1)	77 (35.5)	21 (23.9)	
30.0-39.9 (obese)	137 (44.9)	96 (44.2)	41 (46.6)	
≥40 (extremely obese)	28 (9.2)	17 (7.80)	11 (3.6)	
**Heart disease**	27 (8.8)	18 (8.3)	9 (10.2)	.58
**High blood pressure**	121 (39.7)	93 (42.9)	28 (31.8)	.08
**High cholesterol**	126 (41.3)	95 (43.8)	31 (35.2)	.18
**Asthma**	23 (7.5)	15 (6.9)	8 (9.1)	.51
**Current smoker**	23 (7.5)	15 (6.9)	8 (9.1)	.92

a Significance of difference between completers and noncompleters. Calculated by using the χ^2^ test.

b Spanish compared with "other than Spanish." Calculated by using the Fisher exact test.

c Among participants with diabetes.

**Table 2:** Anthropometric and Laboratory Measures at Baseline, Conclusion, and 3-Month Follow-Up Assessment, Pasos Adelante Program, Douglas, Arizona, 2004-2009

**Characteristic**	Baseline, Mean (SD) (n = 305)	Conclusion, Mean (SD) (n = 255)	3-Month Follow-Up, Mean (SD) (n = 221)
**Anthropomorphic measure**			
Body mass index, kg/m^2^	31.4 (6.2)	31.0 (6.1)	30.9 (6.0)
Waist circumference, cm	95.0 (14.6)	91.2 (13.2)	91.6 (13.0)
Hip circumference, cm	110.7 (13.4)	108.1 (12.1)	107.9 (11.7)
Waist-to-hip ratio	0.86 (0.09)	0.84 (0.08)	0.85 (0.08)
Systolic blood pressure, mm Hg	125.0 (17.0)	121.8 (15.4)	124.9 (17.5)
Diastolic blood pressure, mm Hg	76.9 (9.4)	73.9 (8.6)	75.2 (8.8)
Pulse, beats/30 seconds	34.4 (4.9)	33.9 (4.4)	33.9 (4.4)
**Laboratory measure**			
Total cholesterol, mg/dL	191.6 (41.1)	191.1 (38.2)	193.0 (39.4)
HDL cholesterol, mg/dL	44.6 (12.0)	43.2 (12.1)	45.6 (13.4)
LDL cholesterol, mg/dL	122.2 (37.3)	124.5 (34.1)	123.1 (38.1)
Triglycerides, mg/dL	144.8 (88.4)	148.9 (172.5)	148.7 (96.9)
Glucose, mg/dL	103.0 (43.2)	98.1 (28.4)	99.3 (28.6)

Abbreviations: SD, standard deviation; HDL, high-density lipoprotein; LDL, low-density lipoprotein.

**Table 3:** Anthropometric and Laboratory Characteristics at Conclusion and 3-Month Follow-Up Assessments, Pasos Adelante Program, Douglas, Arizona, 2004-2009

Characteristic	*P* Value^a^	Conclusion (n = 255)		3-Month Follow-Up (n = 221)	
		Estimate (SE)	*P* Value^b^	Estimate (SE)	*P* Value^c^
**Anthropometric measure**					
Body mass index	.009	−0.199 (0.097)	.04	−0.108 (0.104)	.30
Waist circumference	<.001	−3.250 (0.301)	<.001	0.281 (0.321)	.38
Hip circumference	<.001	−2.182 (0.230)	<.001	−0.025 (0.245)	.92
Waist-to-hip ratio	.001	−0.013 (0.004)	<.001	0.004 (0.004)	.34
Systolic blood pressure	.002	−3.208 (0.918)	<.001	2.054 (0.986)	.04
Diastolic blood pressure	<.001	−2.939 (0.557)	<.001	0.789 (0.600)	.19
Pulse	.27	−0.463 (0.348)	.18	−0.054 (0.376)	.89
**Laboratory measure**					
Total cholesterol	.03	−6.897 (2.589)	.008	4.815 (2.786)	.09
Triglycerides	.94	1.448 (8.588)	.87	1.236 (9.254)	.89
HDL cholesterol	.12	−1.105 (0.601	.07	1.229 (0.645)	.06
LDL cholesterol	.24	−3.906 (2.328)	.09	3.165 (2.505)	.21
Glucose	.03	0.686 (1.478)	.64	−4.529 (1.740)	.01

Indicates whether time of assessment was significant. Calculated by using the overall model *F* test.

a Indicates whether time of assessment was significant.

b Compared to baseline, calculated by using the *t* test.

c Compared to conclusion, calculated by using *t* test.
